# 4,4′-[8b,8c-Bis(ethoxycarbonyl)-4,8-dioxo-2,3,5,6-tetra­hydro-1*H*,4*H*-2,3a,4a,6,7a,8a-hexa­azacyclo­penta­[*def*]fluorene-2,6-diyl]dipyridinium bis­(tetra­fluorido­borate)

**DOI:** 10.1107/S1600536808023635

**Published:** 2008-08-06

**Authors:** Shu-Qi Qin, Tao Pang, Yi-Tao Li

**Affiliations:** a College of Chemistry and Engineering, Northwest Normal University, Lanzhou 730070, People’s Republic of China; b School of Chemical and Materials Engineering, Huangshi Institute of Technology, Huangshi 435003, People’s Republic of China

## Abstract

In the title compound, C_26_H_32_N_8_O_6_^2+^·2BF_4_^−^, the cation is built up from four fused rings, *viz*. two nearly planar imidazole rings and two triazine rings exhibiting chair conformations. One eth­oxy group is disordered between two positions in an approximate ratio 3:2. The F atoms of the two anions are each rotationally disordered between two orientations in the same 3:2 ratio. The crystal structure is stabilized by inter­molecular N—H⋯O, C—H⋯F and N—H⋯F inter­actions.

## Related literature

For details of the applications of glycoluril derivatives, see: Wei & Wu (2005 ▶); Wu *et al.* (2002 ▶).
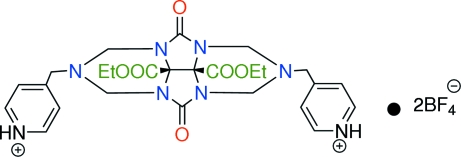


## Experimental

### 

#### Crystal data

C_26_H_32_N_8_O_6_^2+^·2BF_4_^−^
*M* *_r_* = 726.22
Monoclinic, 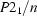

*a* = 17.9399 (17) Å
*b* = 7.9673 (8) Å
*c* = 22.580 (2) Å
β = 93.089 (2)°
*V* = 3222.7 (5) Å^3^
*Z* = 4
Mo *K*α radiation
μ = 0.14 mm^−1^
*T* = 298 (2) K
0.36 × 0.30 × 0.26 mm

#### Data collection

Bruker SMART CCD area-detector diffractometer
Absorption correction: none
17389 measured reflections
5673 independent reflections
3356 reflections with *I* > 2σ(*I*)
*R*_int_ = 0.079

#### Refinement

*R*[*F* ^2^ > 2σ(*F* ^2^)] = 0.066
*wR*(*F* ^2^) = 0.198
*S* = 0.96
5673 reflections
556 parameters
45 restraints
H-atom parameters constrained
Δρ_max_ = 0.39 e Å^−3^
Δρ_min_ = −0.21 e Å^−3^

### 

Data collection: *SMART* (Bruker, 2001 ▶); cell refinement: *SAINT* (Bruker, 2001 ▶); data reduction: *SAINT*; program(s) used to solve structure: *SHELXS97* (Sheldrick, 2008 ▶); program(s) used to refine structure: *SHELXL97* (Sheldrick, 2008 ▶); molecular graphics: *SHELXTL* (Sheldrick, 2008 ▶); software used to prepare material for publication: *SHELXTL*.

## Supplementary Material

Crystal structure: contains datablocks I, global. DOI: 10.1107/S1600536808023635/cv2429sup1.cif

Structure factors: contains datablocks I. DOI: 10.1107/S1600536808023635/cv2429Isup2.hkl

Additional supplementary materials:  crystallographic information; 3D view; checkCIF report

## Figures and Tables

**Table 1:** Hydrogen-bond geometry (Å, °)

*D*—H⋯*A*	*D*—H	H⋯*A*	*D*⋯*A*	*D*—H⋯*A*
C5—H5⋯F7^i^	0.93	2.24	3.124 (6)	158
N1—H1*A*⋯O2^ii^	0.86	2.09	2.825 (4)	143
C1—H1⋯F1′^ii^	0.93	2.16	2.882 (8)	134
C2—H2⋯F3^ii^	0.93	2.42	3.213 (8)	143
C20—H20*B*⋯F8^iii^	0.97	2.29	3.215 (8)	160
C21—H21*B*⋯F8^iii^	0.97	2.47	3.345 (10)	149
C23—H23⋯F5^iii^	0.93	2.33	3.191 (7)	154
C24—H24⋯F3′^iv^	0.93	2.57	3.264 (10)	132
C26—H26⋯F1^v^	0.93	2.48	3.337 (14)	154
C7—H7*B*⋯F4	0.97	2.46	3.273 (9)	142

Symmetry codes: (i) 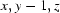; (ii) 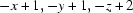; (iii) 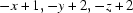; (iv) 
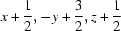
; (v) 
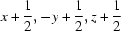
.

**Fig. 1.**
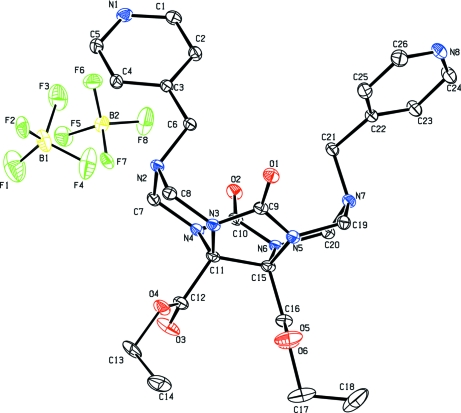
The molecular structure of (I) with 50% probability displacement ellipsoids (arbitrary spheres for H atoms). Only major components of the disordered groups of atoms are shown.

## supplementary crystallographic information

### Comment

Glycoluril derivatives have many areas of applications, such as explosives, slow-release fertilizers, crosslinkers, iodogens, stabilisers of organic compounds against photodegradation, and reagent in combinatorial chemistry (Wu *et al.*, 2002). In continuation of our previous studies in this area (Wei & Wu, 2005), we present the crystal structure of the title compound, (I) (Fig. 1).

All the geometrical parameters for (I) are normal - the distance between the two carbonyl oxygen atoms (O1 and O2) of the glycoluril moiety is 5.238 (5) Å. The distance between the centers of the two pyridyl rings is 7.386 (7) Å. Two pyridyl rings form a dihedral angle of 86.9 (3)°. The crystal packing is stabilized by intermolecular N—H···O, C—H···F and N—H···F interactions (Table 1).

### Experimental

A suspension of di(ethoxycarbonyl) glycoluril (1.43 g, 5 mmol) in 37% aq formaldehyde (3.5 ml) and MeOH (30 ml) was brought to reflux under magnetic stirring. Pyridin-4-ylmethanamine (15 mmol) in MeOH (20 ml) was slowly added dropwise (over 1 h) to the mixture. Then refluxing was continued. The reaction was monitored by TLC. The solvent was removed under reduced pressure and the products were separated by column chromatography (silica gel) with the yield 70%. X-ray quality crystals were grown from a Dichloromethane and Fluoboric acid solution at room temperature.

### Refinement

Positional disorder in the molecule was resolved with occupancies for the major and minor components refining to 0.62 (8) and 0.37 (2) for O4-C13-C14, 0.58 (5) and 0.41 (5) for F1-F2-F3-F4, 0.59 (2) and 0.40 (8) for F5-F6-F7-F8. All H atoms were positioned geometrically (C—H = 0.93–0.97 Å, N—H = 0.86 Å) and refined as riding, allowing for free rotation of methyl groups. The constraint *U*_iso_ (H) = 1.2Ueq (C, N) or 1.5Ueq (methyl C) was applied.

### Crystal data

**Table:**

C_26_H_32_N_8_O_6_^2+^·2BF_4_^–^	*F*_000_ = 1496
*M**_r_* = 726.22	*D*_x_ = 1.497 Mg m^−^^3^
Monoclinic, *P*2_1_/*n*	Mo *K*α radiation λ = 0.71073 Å
Hall symbol: -P 2yn	Cell parameters from 3447 reflections
*a* = 17.9399 (17) Å	θ = 2.7–21.9º
*b* = 7.9673 (8) Å	µ = 0.14 mm^−^^1^
*c* = 22.580 (2) Å	*T* = 298 (2) K
β = 93.089 (2)º	Block, yellow
*V* = 3222.7 (5) Å^3^	0.36 × 0.30 × 0.26 mm
*Z* = 4	

### Data collection

**Table:**

Bruker SMART CCD area-detector diffractometer	3356 reflections with *I* > 2σ(*I*)
Radiation source: fine-focus sealed tube	*R*_int_ = 0.079
Monochromator: graphite	θ_max_ = 25.0º
*T* = 298(2) K	θ_min_ = 1.5º
phi and ω scans	*h* = −20→21
Absorption correction: none	*k* = −9→9
17389 measured reflections	*l* = −26→25
5673 independent reflections	

### Refinement

**Table:**

Refinement on *F*^2^	Secondary atom site location: difference Fourier map
Least-squares matrix: full	Hydrogen site location: inferred from neighbouring sites
*R*[*F*^2^ > 2σ(*F*^2^)] = 0.066	H-atom parameters constrained
*wR*(*F*^2^) = 0.198	*w* = 1/[σ^2^(*F*_o_^2^) + (0.118*P*)^2^] where *P* = (*F*_o_^2^ + 2*F*_c_^2^)/3
*S* = 0.96	(Δ/σ)_max_ < 0.001
5673 reflections	Δρ_max_ = 0.39 e Å^−^^3^
556 parameters	Δρ_min_ = −0.21 e Å^−^^3^
45 restraints	Extinction correction: none
Primary atom site location: structure-invariant direct methods	

### Special details

**Table:**

Geometry. All e.s.d.'s (except the e.s.d. in the dihedral angle between two l.s. planes) are estimated using the full covariance matrix. The cell e.s.d.'s are taken into account individually in the estimation of e.s.d.'s in distances, angles and torsion angles; correlations between e.s.d.'s in cell parameters are only used when they are defined by crystal symmetry. An approximate (isotropic) treatment of cell e.s.d.'s is used for estimating e.s.d.'s involving l.s. planes.
Refinement. Refinement of *F*^2^ against ALL reflections. The weighted *R*-factor *wR* and goodness of fit *S* are based on *F*^2^, conventional *R*-factors *R* are based on *F*, with *F* set to zero for negative *F*^2^. The threshold expression of *F*^2^ > σ(*F*^2^) is used only for calculating *R*-factors(gt) *etc*. and is not relevant to the choice of reflections for refinement. *R*-factors based on *F*^2^ are statistically about twice as large as those based on *F*, and *R*- factors based on ALL data will be even larger.

### Fractional atomic coordinates and isotropic or equivalent isotropic displacement parameters (Å^2^)

**Table:**

	*x*	*y*	*z*	*U*_iso_*/*U*_eq_	Occ. (<1)
C1	0.5001 (2)	0.2921 (4)	1.03044 (15)	0.0681 (9)	
H1	0.4825	0.3271	1.0664	0.082*	
C2	0.57382 (18)	0.3098 (4)	1.01969 (14)	0.0614 (8)	
H2	0.6062	0.3596	1.0480	0.074*	
C3	0.60061 (16)	0.2541 (4)	0.96692 (13)	0.0525 (7)	
C4	0.54954 (19)	0.1868 (5)	0.92583 (15)	0.0774 (11)	
H4	0.5653	0.1498	0.8895	0.093*	
C5	0.4769 (2)	0.1739 (6)	0.93781 (16)	0.0849 (12)	
H5	0.4430	0.1288	0.9095	0.102*	
C6	0.68192 (16)	0.2666 (5)	0.95544 (13)	0.0603 (8)	
H6A	0.7019	0.3691	0.9732	0.072*	
H6B	0.7082	0.1728	0.9744	0.072*	
C7	0.67801 (16)	0.4271 (4)	0.86354 (13)	0.0609 (9)	
H7A	0.6740	0.4110	0.8209	0.073*	
H7B	0.6299	0.4657	0.8758	0.073*	
C8	0.77071 (17)	0.2133 (4)	0.87916 (14)	0.0607 (8)	
H8A	0.7714	0.1844	0.8375	0.073*	
H8B	0.7835	0.1132	0.9020	0.073*	
C9	0.86504 (15)	0.3565 (4)	0.94858 (13)	0.0533 (8)	
C10	0.72970 (16)	0.6712 (4)	0.92212 (12)	0.0544 (8)	
C11	0.81079 (15)	0.5083 (4)	0.86883 (12)	0.0522 (8)	
C12	0.8258 (2)	0.5248 (5)	0.80193 (15)	0.0698 (10)	
O4	0.8874 (7)	0.4592 (18)	0.7910 (3)	0.084 (3)	0.372 (12)
C13	0.9117 (10)	0.4813 (18)	0.7335 (5)	0.102 (5)	0.372 (12)
H13A	0.9653	0.4988	0.7352	0.122*	0.372 (12)
H13B	0.8879	0.5794	0.7154	0.122*	0.372 (12)
C14	0.8922 (18)	0.329 (2)	0.6973 (6)	0.172 (13)	0.372 (12)
H14A	0.8924	0.2319	0.7226	0.258*	0.372 (12)
H14B	0.9283	0.3136	0.6678	0.258*	0.372 (12)
H14C	0.8435	0.3424	0.6781	0.258*	0.372 (12)
C13'	0.8736 (5)	0.3895 (17)	0.7186 (3)	0.119 (4)	0.628 (12)
H13C	0.8497	0.4859	0.6994	0.143*	0.628 (12)
H13D	0.8545	0.2888	0.6989	0.143*	0.628 (12)
C14'	0.9554 (5)	0.3997 (17)	0.7141 (4)	0.135 (5)	0.628 (12)
H14D	0.9719	0.5121	0.7228	0.203*	0.628 (12)
H14E	0.9680	0.3701	0.6747	0.203*	0.628 (12)
H14F	0.9795	0.3235	0.7420	0.203*	0.628 (12)
O4'	0.8572 (4)	0.3859 (10)	0.7797 (2)	0.0828 (19)	0.628 (12)
C15	0.85621 (15)	0.6300 (4)	0.91047 (12)	0.0524 (7)	
C16	0.9183 (2)	0.7291 (5)	0.88022 (16)	0.0695 (9)	
C17	0.9536 (4)	0.9832 (7)	0.8373 (3)	0.150 (2)	
H17A	0.9780	0.9152	0.8084	0.180*	
H17B	0.9277	1.0744	0.8164	0.180*	
C18	1.0099 (4)	1.0520 (9)	0.8811 (4)	0.213 (4)	
H18A	1.0478	0.9691	0.8899	0.320*	
H18B	0.9862	1.0811	0.9168	0.320*	
H18C	1.0324	1.1502	0.8651	0.320*	
C19	0.91213 (16)	0.5955 (5)	1.01245 (13)	0.0619 (9)	
H19A	0.9270	0.5080	1.0406	0.074*	
H19B	0.9550	0.6675	1.0077	0.074*	
C20	0.82271 (18)	0.8131 (4)	0.99351 (13)	0.0599 (8)	
H20A	0.8600	0.8985	0.9873	0.072*	
H20B	0.7798	0.8680	1.0093	0.072*	
C21	0.79600 (17)	0.5878 (5)	1.06348 (12)	0.0634 (9)	
H21A	0.7865	0.4890	1.0392	0.076*	
H21B	0.7496	0.6499	1.0651	0.076*	
C22	0.82226 (16)	0.5353 (4)	1.12506 (13)	0.0562 (8)	
C23	0.8290 (2)	0.6502 (5)	1.17015 (14)	0.0771 (10)	
H23	0.8202	0.7633	1.1623	0.092*	
C24	0.8490 (2)	0.5984 (6)	1.22743 (16)	0.0879 (12)	
H24	0.8535	0.6752	1.2584	0.105*	
C25	0.8385 (2)	0.3710 (5)	1.13763 (16)	0.0801 (10)	
H25	0.8359	0.2917	1.1073	0.096*	
C26	0.8587 (2)	0.3221 (6)	1.19426 (19)	0.0905 (12)	
H26	0.8702	0.2106	1.2028	0.109*	
F1	0.4030 (7)	0.5250 (14)	0.7710 (4)	0.224 (8)	0.585 (14)
F2	0.4475 (4)	0.2852 (6)	0.8070 (3)	0.107 (3)	0.585 (14)
F3	0.3902 (5)	0.4596 (10)	0.8656 (3)	0.159 (4)	0.585 (14)
F4	0.5018 (5)	0.5213 (11)	0.8372 (6)	0.181 (5)	0.585 (14)
F1'	0.4694 (10)	0.5329 (16)	0.8592 (5)	0.179 (7)	0.415 (14)
F2'	0.4928 (11)	0.334 (2)	0.7966 (5)	0.222 (10)	0.415 (14)
F3'	0.4150 (6)	0.5340 (13)	0.7668 (4)	0.106 (4)	0.415 (14)
F4'	0.3802 (7)	0.348 (3)	0.8322 (9)	0.250 (12)	0.415 (14)
F5	0.2541 (3)	0.9974 (8)	0.8457 (3)	0.112 (2)	0.592 (9)
F6	0.2869 (4)	0.8083 (8)	0.9145 (3)	0.130 (3)	0.592 (9)
F7	0.3729 (2)	0.9319 (5)	0.86238 (15)	0.0743 (16)	0.592 (9)
F8	0.3161 (5)	1.0776 (12)	0.9308 (4)	0.168 (5)	0.592 (9)
F5'	0.3113 (7)	0.7954 (11)	0.8864 (7)	0.236 (10)	0.408 (9)
F6'	0.3243 (5)	1.0241 (10)	0.9440 (2)	0.068 (2)	0.408 (9)
F7'	0.3375 (9)	1.047 (3)	0.8487 (3)	0.399 (19)	0.408 (9)
F8'	0.2252 (4)	0.9914 (10)	0.8809 (5)	0.122 (4)	0.408 (9)
B1	0.4375 (3)	0.4445 (7)	0.8168 (2)	0.114 (2)	
B2	0.3046 (2)	0.9579 (6)	0.88888 (19)	0.0864 (14)	
N1	0.45360 (15)	0.2244 (4)	0.98903 (13)	0.0729 (8)	
H1A	0.4072	0.2135	0.9960	0.088*	
N2	0.69559 (13)	0.2664 (3)	0.89217 (10)	0.0581 (7)	
N3	0.82774 (12)	0.3438 (3)	0.89293 (10)	0.0527 (6)	
N4	0.73395 (12)	0.5557 (3)	0.87778 (10)	0.0534 (6)	
N5	0.89045 (13)	0.5179 (3)	0.95504 (10)	0.0543 (7)	
N6	0.79994 (13)	0.7353 (3)	0.93560 (10)	0.0531 (6)	
N7	0.85262 (13)	0.6938 (3)	1.03643 (10)	0.0577 (7)	
N8	0.86144 (19)	0.4371 (5)	1.23672 (13)	0.0894 (10)	
H8	0.8720	0.4045	1.2725	0.107*	
O1	0.87551 (12)	0.2428 (3)	0.98316 (10)	0.0709 (7)	
O2	0.67268 (12)	0.7166 (3)	0.94438 (9)	0.0708 (7)	
O3	0.8013 (2)	0.6342 (5)	0.77298 (12)	0.1221 (12)	
O5	0.97363 (18)	0.6629 (4)	0.86828 (18)	0.1354 (14)	
O6	0.89909 (15)	0.8793 (4)	0.86841 (14)	0.1023 (9)	

### Atomic displacement parameters (Å^2^)

**Table:**

	*U*^11^	*U*^22^	*U*^33^	*U*^12^	*U*^13^	*U*^23^
C1	0.073 (2)	0.074 (2)	0.059 (2)	0.0090 (18)	0.0190 (18)	−0.0043 (17)
C2	0.0612 (19)	0.071 (2)	0.0521 (18)	0.0020 (16)	0.0021 (15)	−0.0069 (16)
C3	0.0543 (17)	0.061 (2)	0.0429 (16)	−0.0001 (14)	0.0046 (13)	0.0026 (14)
C4	0.066 (2)	0.116 (3)	0.0503 (19)	−0.024 (2)	0.0123 (16)	−0.0186 (19)
C5	0.065 (2)	0.130 (4)	0.059 (2)	−0.028 (2)	−0.0003 (18)	−0.006 (2)
C6	0.0514 (17)	0.087 (2)	0.0423 (16)	−0.0085 (16)	0.0040 (13)	0.0037 (15)
C7	0.0502 (17)	0.091 (3)	0.0416 (16)	0.0003 (17)	0.0004 (13)	0.0024 (16)
C8	0.0595 (19)	0.068 (2)	0.0561 (18)	−0.0004 (16)	0.0131 (15)	−0.0044 (16)
C9	0.0419 (15)	0.065 (2)	0.0537 (18)	0.0102 (15)	0.0086 (13)	0.0078 (16)
C10	0.0477 (18)	0.073 (2)	0.0424 (16)	0.0111 (15)	0.0033 (13)	0.0159 (15)
C11	0.0475 (16)	0.071 (2)	0.0394 (15)	0.0035 (14)	0.0119 (12)	0.0059 (14)
C12	0.070 (2)	0.096 (3)	0.0443 (19)	−0.004 (2)	0.0097 (16)	0.010 (2)
O4	0.073 (7)	0.141 (10)	0.041 (4)	0.005 (6)	0.029 (4)	−0.013 (4)
C13	0.109 (11)	0.135 (13)	0.065 (8)	−0.019 (9)	0.037 (8)	−0.029 (7)
C14	0.33 (4)	0.151 (16)	0.047 (9)	−0.120 (18)	0.080 (13)	−0.056 (9)
C13'	0.155 (9)	0.171 (12)	0.037 (5)	0.000 (8)	0.043 (5)	−0.027 (6)
C14'	0.131 (8)	0.181 (12)	0.100 (7)	0.013 (7)	0.058 (6)	0.019 (7)
O4'	0.084 (4)	0.116 (5)	0.051 (3)	0.008 (3)	0.024 (3)	−0.009 (3)
C15	0.0444 (15)	0.068 (2)	0.0463 (16)	0.0054 (14)	0.0121 (12)	0.0079 (14)
C16	0.066 (2)	0.077 (3)	0.067 (2)	0.0007 (19)	0.0210 (17)	0.0095 (19)
C17	0.131 (4)	0.119 (4)	0.207 (7)	−0.017 (4)	0.073 (5)	0.059 (4)
C18	0.157 (6)	0.196 (7)	0.298 (10)	−0.108 (6)	0.110 (7)	−0.109 (7)
C19	0.0492 (17)	0.082 (2)	0.0541 (18)	−0.0028 (16)	−0.0047 (14)	0.0069 (17)
C20	0.0609 (18)	0.068 (2)	0.0511 (18)	0.0042 (16)	0.0077 (14)	0.0024 (16)
C21	0.0565 (18)	0.090 (3)	0.0429 (16)	−0.0060 (17)	−0.0017 (13)	0.0052 (16)
C22	0.0568 (18)	0.066 (2)	0.0456 (17)	−0.0060 (15)	0.0015 (13)	0.0033 (16)
C23	0.102 (3)	0.077 (3)	0.051 (2)	0.005 (2)	−0.0010 (18)	0.0020 (18)
C24	0.120 (3)	0.090 (3)	0.052 (2)	0.002 (3)	−0.005 (2)	−0.002 (2)
C25	0.098 (3)	0.077 (3)	0.064 (2)	−0.010 (2)	−0.0044 (19)	0.0000 (19)
C26	0.109 (3)	0.076 (3)	0.085 (3)	−0.003 (2)	−0.012 (2)	0.019 (2)
F1	0.336 (19)	0.150 (13)	0.173 (12)	0.027 (11)	−0.087 (12)	−0.006 (10)
F2	0.159 (6)	0.071 (3)	0.085 (4)	−0.006 (3)	−0.032 (4)	−0.028 (2)
F3	0.193 (9)	0.149 (6)	0.136 (5)	0.006 (5)	0.004 (5)	−0.070 (4)
F4	0.154 (6)	0.135 (6)	0.243 (12)	0.009 (5)	−0.098 (8)	−0.046 (7)
F1'	0.232 (18)	0.161 (10)	0.140 (8)	−0.009 (11)	−0.036 (9)	−0.106 (7)
F2'	0.34 (2)	0.216 (16)	0.103 (7)	0.143 (15)	−0.038 (12)	−0.037 (9)
F3'	0.142 (8)	0.093 (9)	0.080 (7)	0.024 (6)	−0.034 (6)	−0.010 (6)
F4'	0.162 (11)	0.31 (2)	0.28 (2)	−0.112 (15)	−0.040 (13)	0.107 (18)
F5	0.102 (4)	0.128 (4)	0.102 (4)	0.020 (3)	−0.023 (3)	0.024 (3)
F6	0.100 (4)	0.181 (7)	0.105 (4)	−0.050 (4)	−0.017 (3)	0.065 (4)
F7	0.088 (3)	0.096 (3)	0.040 (2)	0.007 (2)	0.0069 (17)	−0.0170 (18)
F8	0.079 (4)	0.241 (10)	0.185 (8)	−0.010 (6)	0.017 (5)	−0.132 (8)
F5'	0.176 (14)	0.169 (13)	0.35 (2)	0.089 (11)	−0.129 (14)	−0.149 (14)
F6'	0.078 (5)	0.087 (4)	0.040 (3)	−0.001 (3)	0.019 (3)	−0.005 (3)
F7'	0.38 (2)	0.75 (4)	0.066 (5)	−0.40 (3)	−0.036 (9)	0.084 (13)
F8'	0.115 (6)	0.096 (5)	0.152 (9)	−0.011 (4)	−0.041 (6)	−0.016 (5)
B1	0.144 (6)	0.121 (6)	0.073 (4)	0.010 (5)	−0.030 (4)	−0.020 (4)
B2	0.075 (3)	0.104 (4)	0.079 (3)	−0.022 (3)	−0.010 (3)	0.001 (3)
N1	0.0493 (15)	0.094 (2)	0.076 (2)	−0.0020 (14)	0.0089 (14)	0.0145 (17)
N2	0.0514 (14)	0.0809 (19)	0.0426 (14)	−0.0030 (13)	0.0077 (11)	0.0029 (12)
N3	0.0486 (13)	0.0657 (17)	0.0446 (13)	0.0046 (12)	0.0099 (10)	0.0045 (12)
N4	0.0462 (14)	0.0745 (18)	0.0398 (13)	0.0085 (12)	0.0047 (10)	0.0058 (12)
N5	0.0453 (13)	0.0681 (18)	0.0495 (14)	0.0053 (12)	0.0027 (11)	0.0071 (12)
N6	0.0493 (14)	0.0675 (17)	0.0432 (13)	0.0062 (12)	0.0081 (10)	0.0060 (11)
N7	0.0521 (14)	0.0757 (18)	0.0452 (14)	0.0003 (13)	0.0016 (11)	0.0054 (13)
N8	0.110 (3)	0.104 (3)	0.0519 (18)	−0.011 (2)	−0.0121 (16)	0.0194 (19)
O1	0.0749 (15)	0.0727 (16)	0.0645 (14)	0.0119 (12)	−0.0024 (11)	0.0169 (12)
O2	0.0494 (12)	0.1031 (19)	0.0607 (13)	0.0187 (12)	0.0108 (10)	−0.0037 (12)
O3	0.174 (3)	0.138 (3)	0.0563 (16)	0.045 (2)	0.0260 (18)	0.0357 (18)
O5	0.084 (2)	0.127 (3)	0.202 (4)	0.016 (2)	0.079 (2)	0.053 (3)
O6	0.0888 (18)	0.091 (2)	0.131 (2)	−0.0037 (16)	0.0438 (17)	0.0360 (18)

### Geometric parameters (Å, °)

**Table:**

C1—N1	1.334 (4)	C15—C16	1.553 (4)
C1—C2	1.364 (5)	C16—O5	1.169 (4)
C1—H1	0.9300	C16—O6	1.269 (4)
C2—C3	1.382 (4)	C17—C18	1.482 (9)
C2—H2	0.9300	C17—O6	1.487 (5)
C3—C4	1.377 (4)	C17—H17A	0.9700
C3—C6	1.499 (4)	C17—H17B	0.9700
C4—C5	1.348 (5)	C18—H18A	0.9600
C4—H4	0.9300	C18—H18B	0.9600
C5—N1	1.314 (5)	C18—H18C	0.9600
C5—H5	0.9300	C19—N7	1.452 (4)
C6—N2	1.462 (4)	C19—N5	1.470 (4)
C6—H6A	0.9700	C19—H19A	0.9700
C6—H6B	0.9700	C19—H19B	0.9700
C7—N4	1.458 (4)	C20—N7	1.441 (4)
C7—N2	1.461 (4)	C20—N6	1.485 (4)
C7—H7A	0.9700	C20—H20A	0.9700
C7—H7B	0.9700	C20—H20B	0.9700
C8—N2	1.457 (4)	C21—N7	1.478 (4)
C8—N3	1.480 (4)	C21—C22	1.503 (4)
C8—H8A	0.9700	C21—H21A	0.9700
C8—H8B	0.9700	C21—H21B	0.9700
C9—O1	1.205 (3)	C22—C25	1.368 (5)
C9—N5	1.369 (4)	C22—C23	1.370 (5)
C9—N3	1.395 (4)	C23—C24	1.386 (5)
C10—O2	1.219 (3)	C23—H23	0.9300
C10—N4	1.365 (4)	C24—N8	1.319 (5)
C10—N6	1.379 (4)	C24—H24	0.9300
C11—N3	1.445 (4)	C25—C26	1.367 (5)
C11—N4	1.454 (4)	C25—H25	0.9300
C11—C15	1.551 (4)	C26—N8	1.325 (5)
C11—C12	1.554 (4)	C26—H26	0.9300
C12—O3	1.161 (4)	F1—B1	1.341 (7)
C12—O4	1.258 (11)	F2—B1	1.302 (7)
C12—O4'	1.351 (8)	F3—B1	1.431 (7)
O4—C13	1.402 (9)	F4—B1	1.363 (7)
C13—C14	1.497 (10)	F1'—B1	1.296 (8)
C13—H13A	0.9700	F2'—B1	1.418 (8)
C13—H13B	0.9700	F3'—B1	1.379 (7)
C14—H14A	0.9600	F4'—B1	1.345 (8)
C14—H14B	0.9600	F5—B2	1.332 (5)
C14—H14C	0.9600	F6—B2	1.370 (6)
C13'—O4'	1.427 (7)	F7—B2	1.409 (5)
C13'—C14'	1.480 (9)	F8—B2	1.352 (6)
C13'—H13C	0.9700	F5'—B2	1.301 (8)
C13'—H13D	0.9700	F6'—B2	1.380 (7)
C14'—H14D	0.9600	F7'—B2	1.319 (7)
C14'—H14E	0.9600	F8'—B2	1.451 (7)
C14'—H14F	0.9600	N1—H1A	0.8600
C15—N6	1.451 (4)	N8—H8	0.8600
C15—N5	1.456 (4)		
			
N1—C1—C2	119.5 (3)	H20A—C20—H20B	107.8
N1—C1—H1	120.2	N7—C21—C22	110.7 (2)
C2—C1—H1	120.2	N7—C21—H21A	109.5
C1—C2—C3	120.4 (3)	C22—C21—H21A	109.5
C1—C2—H2	119.8	N7—C21—H21B	109.5
C3—C2—H2	119.8	C22—C21—H21B	109.5
C4—C3—C2	117.2 (3)	H21A—C21—H21B	108.1
C4—C3—C6	121.9 (3)	C25—C22—C23	118.4 (3)
C2—C3—C6	121.0 (3)	C25—C22—C21	120.7 (3)
C5—C4—C3	120.6 (3)	C23—C22—C21	120.8 (3)
C5—C4—H4	119.7	C22—C23—C24	120.2 (4)
C3—C4—H4	119.7	C22—C23—H23	119.9
N1—C5—C4	120.7 (3)	C24—C23—H23	119.9
N1—C5—H5	119.7	N8—C24—C23	118.1 (4)
C4—C5—H5	119.7	N8—C24—H24	120.9
N2—C6—C3	112.6 (2)	C23—C24—H24	120.9
N2—C6—H6A	109.1	C26—C25—C22	120.6 (4)
C3—C6—H6A	109.1	C26—C25—H25	119.7
N2—C6—H6B	109.1	C22—C25—H25	119.7
C3—C6—H6B	109.1	N8—C26—C25	118.5 (4)
H6A—C6—H6B	107.8	N8—C26—H26	120.7
N4—C7—N2	112.9 (2)	C25—C26—H26	120.7
N4—C7—H7A	109.0	F1'—B1—F2	126.6 (8)
N2—C7—H7A	109.0	F1'—B1—F1	118.5 (11)
N4—C7—H7B	109.0	F2—B1—F1	113.4 (6)
N2—C7—H7B	109.0	F1'—B1—F4'	115.7 (8)
H7A—C7—H7B	107.8	F2—B1—F4'	66.5 (10)
N2—C8—N3	112.9 (2)	F1—B1—F4'	98.4 (9)
N2—C8—H8A	109.0	F1'—B1—F4	34.4 (7)
N3—C8—H8A	109.0	F2—B1—F4	111.9 (6)
N2—C8—H8B	109.0	F1—B1—F4	113.1 (7)
N3—C8—H8B	109.0	F4'—B1—F4	144.5 (8)
H8A—C8—H8B	107.8	F1'—B1—F3'	115.0 (8)
O1—C9—N5	126.7 (3)	F2—B1—F3'	113.7 (7)
O1—C9—N3	125.4 (3)	F1—B1—F3'	10.4 (10)
N5—C9—N3	107.8 (3)	F4'—B1—F3'	108.2 (6)
O2—C10—N4	125.7 (3)	F4—B1—F3'	104.5 (8)
O2—C10—N6	125.2 (3)	F1'—B1—F2'	106.6 (7)
N4—C10—N6	109.0 (2)	F2—B1—F2'	40.3 (9)
N3—C11—N4	111.4 (2)	F1—B1—F2'	110.4 (9)
N3—C11—C15	104.2 (2)	F4'—B1—F2'	106.7 (8)
N4—C11—C15	103.0 (2)	F4—B1—F2'	78.0 (8)
N3—C11—C12	113.5 (3)	F3'—B1—F2'	103.6 (7)
N4—C11—C12	109.2 (2)	F1'—B1—F3	69.1 (7)
C15—C11—C12	115.0 (3)	F2—B1—F3	107.8 (5)
O3—C12—O4	120.7 (6)	F1—B1—F3	106.6 (7)
O3—C12—O4'	124.0 (4)	F4'—B1—F3	49.9 (9)
O4—C12—O4'	36.7 (5)	F4—B1—F3	103.2 (5)
O3—C12—C11	122.0 (4)	F3'—B1—F3	115.3 (7)
O4—C12—C11	110.9 (5)	F2'—B1—F3	139.0 (9)
O4'—C12—C11	112.9 (4)	F5'—B2—F7'	117.6 (8)
C12—O4—C13	116.8 (11)	F5'—B2—F5	105.4 (7)
O4—C13—C14	109.2 (9)	F7'—B2—F5	71.4 (6)
O4—C13—H13A	109.8	F5'—B2—F8	136.1 (8)
C14—C13—H13A	109.8	F7'—B2—F8	92.5 (8)
O4—C13—H13B	109.8	F5—B2—F8	114.7 (6)
C14—C13—H13B	109.8	F5'—B2—F6	34.6 (8)
H13A—C13—H13B	108.3	F7'—B2—F6	152.2 (9)
O4'—C13'—C14'	108.9 (7)	F5—B2—F6	110.6 (5)
O4'—C13'—H13C	109.9	F8—B2—F6	110.3 (6)
C14'—C13'—H13C	109.9	F5'—B2—F6'	113.5 (7)
O4'—C13'—H13D	109.9	F7'—B2—F6'	108.1 (6)
C14'—C13'—H13D	109.9	F5—B2—F6'	134.5 (6)
H13C—C13'—H13D	108.3	F8—B2—F6'	22.6 (6)
C13'—C14'—H14D	109.5	F6—B2—F6'	90.3 (5)
C13'—C14'—H14E	109.5	F5'—B2—F7	75.6 (8)
H14D—C14'—H14E	109.5	F7'—B2—F7	49.8 (10)
C13'—C14'—H14F	109.5	F5—B2—F7	107.3 (4)
H14D—C14'—H14F	109.5	F8—B2—F7	107.3 (5)
H14E—C14'—H14F	109.5	F6—B2—F7	106.2 (5)
C12—O4'—C13'	117.0 (7)	F6'—B2—F7	104.6 (5)
N6—C15—N5	111.1 (2)	F5'—B2—F8'	105.7 (6)
N6—C15—C11	104.2 (2)	F7'—B2—F8'	106.7 (7)
N5—C15—C11	102.9 (2)	F5—B2—F8'	40.6 (4)
N6—C15—C16	114.1 (3)	F8—B2—F8'	94.0 (6)
N5—C15—C16	109.3 (2)	F6—B2—F8'	88.0 (5)
C11—C15—C16	114.6 (2)	F6'—B2—F8'	104.1 (6)
O5—C16—O6	126.9 (3)	F7—B2—F8'	147.7 (6)
O5—C16—C15	120.7 (4)	C5—N1—C1	121.6 (3)
O6—C16—C15	112.3 (3)	C5—N1—H1A	119.2
C18—C17—O6	109.4 (6)	C1—N1—H1A	119.2
C18—C17—H17A	109.8	C8—N2—C7	110.2 (2)
O6—C17—H17A	109.8	C8—N2—C6	113.8 (2)
C18—C17—H17B	109.8	C7—N2—C6	112.8 (3)
O6—C17—H17B	109.8	C9—N3—C11	110.8 (2)
H17A—C17—H17B	108.2	C9—N3—C8	122.2 (2)
C17—C18—H18A	109.5	C11—N3—C8	115.4 (2)
C17—C18—H18B	109.5	C10—N4—C11	111.6 (2)
H18A—C18—H18B	109.5	C10—N4—C7	124.8 (2)
C17—C18—H18C	109.5	C11—N4—C7	115.7 (2)
H18A—C18—H18C	109.5	C9—N5—C15	112.1 (2)
H18B—C18—H18C	109.5	C9—N5—C19	124.0 (3)
N7—C19—N5	113.0 (2)	C15—N5—C19	115.7 (3)
N7—C19—H19A	109.0	C10—N6—C15	110.3 (2)
N5—C19—H19A	109.0	C10—N6—C20	123.7 (2)
N7—C19—H19B	109.0	C15—N6—C20	114.9 (2)
N5—C19—H19B	109.0	C20—N7—C19	111.1 (2)
H19A—C19—H19B	107.8	C20—N7—C21	114.6 (2)
N7—C20—N6	113.1 (3)	C19—N7—C21	112.4 (3)
N7—C20—H20A	109.0	C24—N8—C26	124.0 (3)
N6—C20—H20A	109.0	C24—N8—H8	118.0
N7—C20—H20B	109.0	C26—N8—H8	118.0
N6—C20—H20B	109.0	C16—O6—C17	116.6 (3)

### Hydrogen-bond geometry (Å, °)

**Table:**

*D*—H···*A*	*D*—H	H···*A*	*D*···*A*	*D*—H···*A*
C5—H5···F7^i^	0.93	2.24	3.124 (6)	158
N1—H1A···O2^ii^	0.86	2.09	2.825 (4)	143
C1—H1···F1'^ii^	0.93	2.16	2.882 (8)	134
C2—H2···F3^ii^	0.93	2.42	3.213 (8)	143
C20—H20B···F8^iii^	0.97	2.29	3.215 (8)	160
C21—H21B···F8^iii^	0.97	2.47	3.345 (10)	149
C23—H23···F5^iii^	0.93	2.33	3.191 (7)	154
C24—H24···F3'^iv^	0.93	2.57	3.264 (10)	132
C26—H26···F1^v^	0.93	2.48	3.337 (14)	154
C7—H7B···F4	0.97	2.46	3.273 (9)	142

Symmetry codes: (i) *x*, *y*−1, *z*; (ii) −*x*+1, −*y*+1, −*z*+2; (iii) −*x*+1, −*y*+2, −*z*+2; (iv) *x*+1/2, −*y*+3/2, *z*+1/2; (v) *x*+1/2, −*y*+1/2, *z*+1/2.
